# Pseudoprogression in advanced non-small cell lung cancer treated with combination chemoimmunotherapy: a case report

**DOI:** 10.1186/s13256-022-03485-6

**Published:** 2022-07-25

**Authors:** Amrit S. Gonugunta, Mitchell S. von Itzstein, David E. Gerber

**Affiliations:** 1 School of Medicine, UT Southwestern Medical Center, Dallas, TX USA; 2 Division of Hematology and Oncology, Department of Internal Medicine, UT Southwestern Medical Center, 5323 Harry Hines Blvd., Mail Code 8852, Dallas, TX 75390-8852 USA; 3 Harold C. Simmons Comprehensive Cancer Center, UT Southwestern Medical Center, Dallas, TX USA; 4 Department of Population and Data Sciences, UT Southwestern Medical Center, Dallas, TX USA

**Keywords:** Checkpoint inhibitor, Immunotherapy, Pseudoprogression, Response

## Abstract

**Background:**

Pseudoprogression, the initial apparent worsening of cancer prior to eventual improvement, is a documented feature of immune checkpoint inhibitor administration and presents a challenge to clinicians distinguishing true progression from pseudoprogression. This phenomenon does not typically occur with traditional cytotoxic chemotherapy. We present a case in which a patient treated with combination carboplatin-pemetrexed plus pembrolizumab experienced transient radiographic worsening of disease with subsequent regression.

**Case presentation:**

A 65-year-old never-smoking white male with advanced sarcomatoid non-small cell lung cancer (NSCLC) harboring a *MET* exon 14 skipping mutation and with PD-L1 tumor proportion score of 80% was initiated on combination chemotherapy plus immune checkpoint inhibitor (ICI) therapy after progression on a MET inhibitor. After two cycles of carboplatin-pemetrexed plus pembrolizumab, repeat imaging suggested disease progression. Following discontinuation of the carboplatin-pemetrexed plus pembrolizumab regimen, the patient reported improved symptoms and energy levels, which were attributed to the waning of treatment-associated toxicities. On the day prior to initiation of the next planned line of therapy, repeat imaging was preformed to provide a baseline for treatment efficacy. Imaging revealed improvement compared to the prior imaging. Chemotherapy with carboplatin-pemetrexed plus pembrolizumab was resumed, with response ongoing 8 months later.

**Conclusions:**

Pseudoprogression is a documented feature of ICI administration. Pseudoprogression is not typically observed in patients treated with traditional cytotoxic chemotherapy and has not yet been documented in patients treated with combination cytotoxic chemotherapy plus immunotherapy. At this time, there are no reliable means to predict or diagnose these rare events; therefore, more studies should be conducted to understand which patients are predisposed to developing this phenomenon and to increase clinical recognition.

## Background

With a novel mechanism of action and unprecedented clinical efficacy in multiple tumor types, immune checkpoint inhibitors (ICI) have revolutionized cancer therapy. Yet these promising treatments have also introduced new challenges. Compared to the well-characterized and predictable toxicities of cytotoxic chemotherapy or molecularly targeted therapies, ICI-induced immune-related adverse events (irAE) affect a far broader range of organ systems, may mimic other clinical conditions, and may occur at almost any point in treatment. Clinical characteristics generally not considered relevant to other cancer treatments, such as body mass index, history of autoimmune disease, and exposure to antibiotics, may influence both the efficacy and tolerability of ICI treatment [1–4].

Perhaps one of the most disconcerting features of ICI administration is the potential for pseudoprogression. While early changes in tumor dimensions reliably indicate the benefits of chemotherapy and targeted therapy, in a subset of patients treated with ICI transient radiographic worsening may precede subsequent benefit [5]. Some studies indicate that pseudoprogression may occur in up to 10% of patients receiving ICI therapy [6].

In advanced non-small cell lung cancer (NSCLC), the combination of anti-programmed death 1 (PD-1) plus chemotherapy or the combination of anti-PD-1 and anti-cytotoxic T lymphocyte antigen 4 (CTLA4) plus chemotherapy offers the opportunity to deliver fast-acting cytotoxic agents along with slower but potentially more durably acting ICI. However, as demonstrated in the case presented here, pseudoprogression may still occur with such regimens.

 This study was approved by the UT Southwestern Institutional Review Board (IRB #STU 082015-053).

## Case presentation

A 65-year-old never-smoking white male with advanced sarcomatoid NSCLC harboring a *MET* exon 14 skipping mutation and with a PD-L1 tumor proportion score of 80% was initiated on combination chemotherapy plus ICI therapy after progression on a MET inhibitor. At the time, positron emission tomography (PET)-computed tomography (CT) demonstrated diffuse metastases in the liver, adrenal gland, and bones. Despite high tumor PD-L1 expression, the decision was made to initiate combination chemotherapy plus ICI rather than ICI monotherapy due to the patient’s never-smoking status.

The patient received two cycles of carboplatin-pemetrexed plus pembrolizumab. He tolerated treatment well, with no high-grade toxicities. However, repeat imaging after two cycles demonstrated concern for disease progression, with enlarging lesions in the lung, liver, adrenal gland, neck, and skull. Although clear growth was observed, it did not meet the threshold for hyperprogression, which is most commonly defined as at least a doubling of tumor growth rate [7, 8]. Given primary disease progression and the high burden of disease, the clinical team planned to change treatment to docetaxel plus ramucirumab. In the weeks following discontinuation of the carboplatin-pemetrexed plus pembrolizumab regimen, the patient reported improved symptoms and energy level, which were attributed to waning of treatment-associated toxicities.

**Fig. 1:**
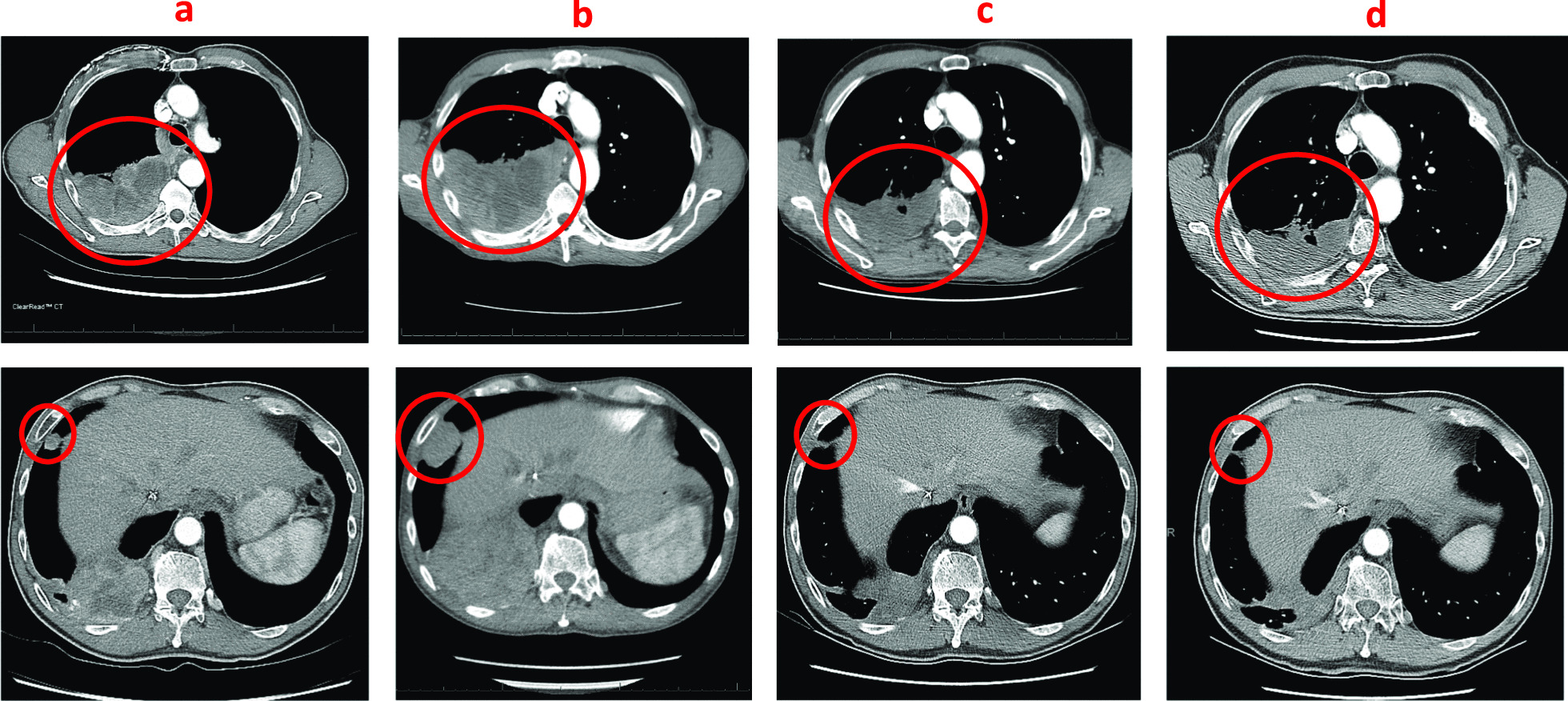
Serial computed tomography axial images. **a** Baseline, **b** after 2 cycles of chemotherapy + immune checkpoint inhibitor (ICI), **c** after 4 cycles of chemotherapy + ICI and **d** 4 cycles of maintenance ICI. Circled regions denote right-sided thoracic tumors

**Fig. 2:**
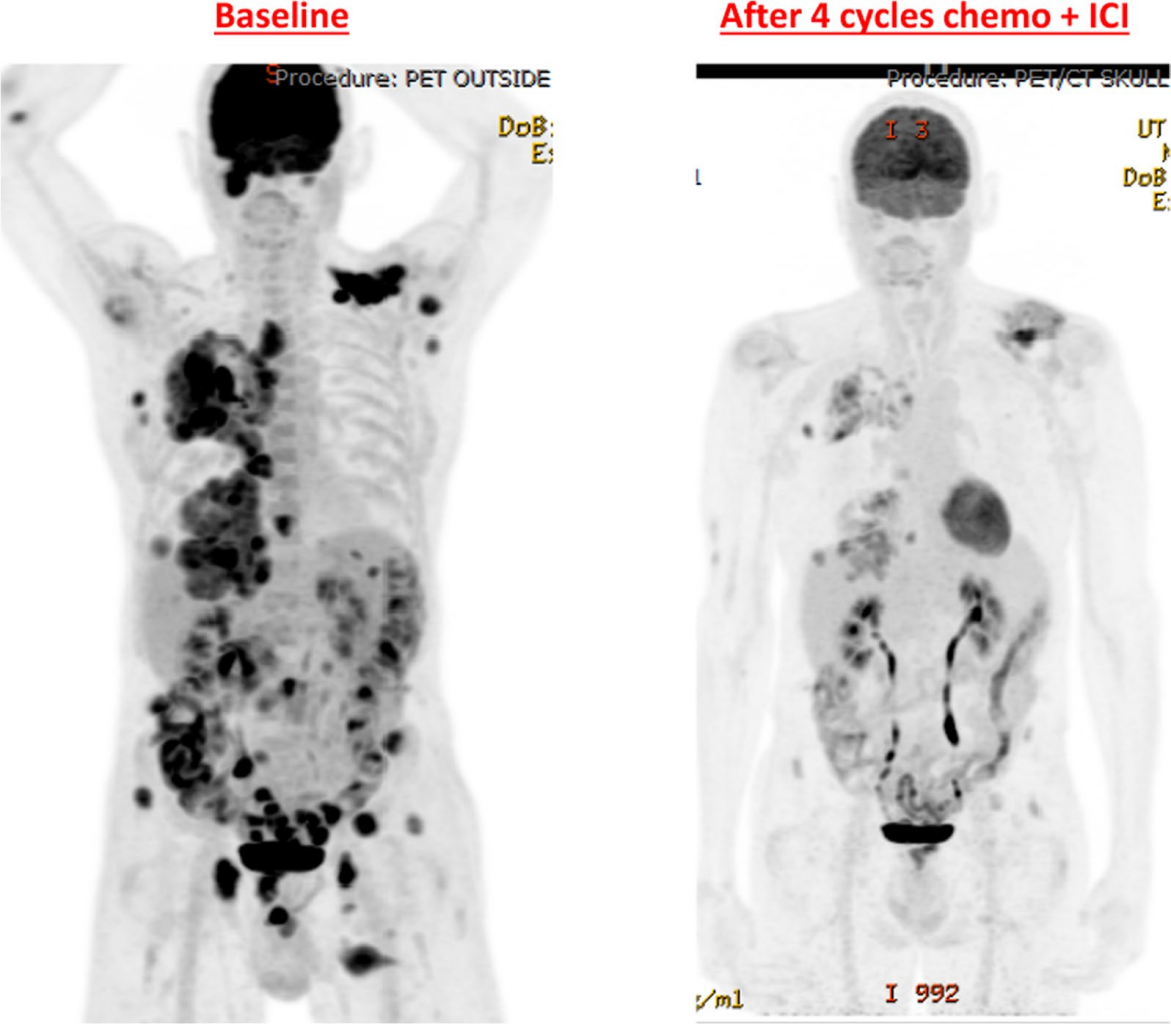
Serial positron emission tomography coronal images. **a** Baseline, **b** after 4 cycles of chemotherapy + ICI

On the day prior to the planned initiation of docetaxel plus ramucirumab, repeat imaging was performed to provide a near-term baseline for subsequent efficacy assessment. The chest CT demonstrated improvement compared to the prior CT image. Accordingly, instead of changing the treatment regimen, the clinical team proceeded with two additional cycles of carboplatin-pemetrexed plus pembrolizumab, following which further response was noted on the chest CT and PET images (Figs. 1, 2). Subsequently, maintenance therapy was initiated with pemetrexed plus pembrolizumab, which was changed to pembrolizumab monotherapy after one cycle due to serum creatinine elevation and fatigue. Since then, the patient has received six cycles of maintenance pembrolizumab, and disease control remains ongoing at 6 months after the initiation of combination chemotherapy plus ICI. Aside from requiring a mild levothyroxine dose adjustment, the patient has had no substantial toxicities.

## Discussion and conclusions

To our knowledge, this article represents the first reported case of pseudoprogression in a patient treated with combination chemotherapy plus ICI. With ICI alone, the reported incidence of pseudoprogression is 2–6% [9, 10]. How to distinguish these cases remains an ongoing area of research. While it has been suggested that clinical symptom trends (worsening in true progression vs. stable or improving in pseudoprogression) may distinguish these processes, it is not clear how reliable this strategy is. Indeed, pseudoprogression has been recognized in patients months after clinical deterioration, treatment cessation, and hospice referral [11]. PET-CT images, both of which document inflammatory changes and neoplastic tissue, routinely demonstrate fluorodeoxyglucose (FDG) uptake but may also fail to differentiate pseudoprogression and true progression [12]. If feasible, biopsy of an enlarging lesion may provide insight, with pseudoprogression featuring CD3, CD4, and CD8 T cell lymphoid infiltrates [13]. More convenient than tissue sampling, cell-free DNA (cfDNA) burden kinetics as a means to ascertain pseudoprogression represents an area of active investigation.

Concern for pseudoprogression resulted in specific recommendations for radiographic monitoring of ICI efficacy. In contrast to conventional response evaluation criteria in solid tumors (RECIST), immune-related response criteria (irRC) incorporate moderate enlargement of pre-existing lesions and appearance of new lesions into the domain of disease control [14]. However, recent NSCLC clinical trials of combination chemotherapy plus ICI have employed standard RECIST [15, 16]. Additionally, these trials perform and act on early radiographic efficacy assessments (typically after 2 cycles, approximately 6 weeks after treatment initiation), in contrast to the later and confirmatory assessments recommended for immunotherapy [17].

If pseudoprogression occurs in a patient treated with combination chemotherapy plus ICI, does this imply chemotherapy resistance? Alternatively, chemotherapy might have efficacy, but radiographic manifestation of immune cell influx outweighs that of tumor regression, resulting in tumor enlargement on imaging studies. In this case, while combination carboplatin-pemetrexed was continued for two more cycles and single-agent pemetrexed for one cycle of maintenance therapy thereafter, ICI monotherapy with pembrolizumab has resulted in months of ongoing disease control. For now, whether cytotoxic agents should be discontinued due to potential lack of efficacy when pseudoprogression occurs with combination chemoimmunotherapy will need to be considered on a case-by-case basis.

How to determine which cases are experiencing true progression versus pseudoprogression, which is attributed to an early influx of immune cells resulting in apparent tumor enlargement, represents a true challenge for clinicians [18]. Ideally, all patients could continue ICI after initial progression. Such an approach would not only ensure subsequent pseudoprogression and clinical benefit are not missed, but would also provide insight into the true incidence of this phenomenon. In reality, however, many patients cannot afford to continue potentially ineffective treatment in the setting of advanced malignancy.

To our knowledge, this is the first reported case of pseudoprogression with combination chemoimmunotherapy, which serves as an important reminder that this rare phenomenon may occur in various clinical contexts. Indeed, the potential for early disease control from cytotoxic agents has served as the rationale for such regimens as ipilimumab + nivolumab combined with two initial cycles of platinum doublet chemotherapy [19]. Currently, there is no reliable means to predict or diagnose these rare events. Given the potential long-term benefits of ICI therapy and the potential harms of unchallenged cancer growth, clinicians face high stakes when choosing whether to continue or change treatment. This is all the more true when one considers that pseudoprogression has been associated with improved survival [20]. There remains a clear need for practical and accurate means to recognize pseudoprogression. This case report represents one novel presentation of pseudoprogression with combination chemotherapy with immunotherapy. There may be other presentations of this phenomenon; therefore, more studies, analyzing why presentations like this exist should be conducted.

## Data Availability

Data are available on reasonable request to corresponding author.

## Declarations

### Declarations

Ethics approval and consent to participate

This study was approved by the UT Southwestern Institutional Review Board (IRB #STU 082015-053).

## References

[1] von Itzstein MS, Gonugunta AS, Wang Y, *et al.* Divergent prognostic effects of pre-existing and treatment-emergent thyroid dysfunction in patients treated with immune checkpoint inhibitors. Cancer Immunol Immunother. 2022. 10.1007/s00262-022-03151-2. PMC9308834 35072744

[2] von Itzstein MS, Gonugunta AS, Sheffield T, *et al*. Association between antibiotic exposure and systemic immune parameters in cancer patients receiving checkpoint inhibitor therapy. Cancers (Basel). 2022;14(5):1327. 10.3390/cancers14051327 PMC8909108 35267634

[3] Ahmed M, von Itzstein MS, Sheffield T, *et al.* Association between body mass index, dosing strategy, and efficacy of immune checkpoint inhibitors. J Immunother Cancer. 2021;9(6):e002349. 10.1136/jitc-2021-002349 PMC8237749 34127546

[4] von Itzstein MS, Gonugunta AS, Mayo HG, Minna JD, Gerber DE. Immunotherapy use in patients with lung cancer and comorbidities. Cancer J. 2020;26(6):525–36. 10.1097/PPO.0000000000000484 PMC7735252 33298724

[5] Wang Q, Gao J, Wu X. Pseudoprogression and hyperprogression after checkpoint blockade. Int Immunopharmacol. 2018;58:125–35. 10.1016/j.intimp.2018.03.018 29579717

[6] Borcoman E, Kanjanapan Y, Champiat S, *et al*. Novel patterns of response under immunotherapy. Ann Oncol. 2019;30(3):385–96. 10.1093/annonc/mdz003 30657859

[7] Popat V, Gerber DE. Hyperprogressive disease: a distinct effect of immunotherapy? J Thorac Dis. 2019;11(Suppl 3):S262–5. 10.21037/jtd.2019.01.97 PMC6424758 30997192

[8] Champiat S, Dercle L, Ammari S, *et al*. Hyperprogressive disease is a new pattern of progression in cancer patients treated by anti-PD-1/PD-L1. Clin Cancer Res. 2017;23(8):1920–8. 10.1158/1078-0432.CCR-16-1741 27827313

[9] Fujimoto D, Yoshioka H, Kataoka Y, *et al*. Pseudoprogression in previously treated patients with non-small cell lung cancer who received nivolumab monotherapy. J Thorac Oncol. 2019;14(3):468–74. 10.1016/j.jtho.2018.10.167 30468872

[10] Kim HK, Heo MH, Lee HS, *et al*. Comparison of RECIST to immune-related response criteria in patients with non-small cell lung cancer treated with immune-checkpoint inhibitors. Cancer Chemother Pharmacol. 2017;80(3):591–8. 10.1007/s00280-017-3396-4 28733892

[11] Elias R, Kapur P, Pedrosa I, Brugarolas J. Renal cell carcinoma pseudoprogression with clinical deterioration: to hospice and back. Clin Genitourin Cancer. 2018;16(6):485–8. 10.1016/j.clgc.2018.07.015 PMC7085275 30100272

[12] Sachpekidis C, Larribere L, Pan L, Haberkorn U, Dimitrakopoulou-Strauss A, Hassel JC. Predictive value of early 18F-FDG PET/CT studies for treatment response evaluation to ipilimumab in metastatic melanoma: preliminary results of an ongoing study. Eur J Nucl Med Mol Imaging. 2015;42(3):386–96. 10.1007/s00259-014-2944-y 25359635

[13] Ma Y, Wang Q, Dong Q, Zhan L, Zhang J. How to differentiate pseudoprogression from true progression in cancer patients treated with immunotherapy. Am J Cancer Res. 2019;9(8):1546–53. PMC6726978 31497342

[14] Hodi FS, Ballinger M, Lyons B, *et al*. Immune-modified response evaluation criteria in solid tumors (imRECIST): refining Guidelines to Assess the Clinical Benefit of Cancer Immunotherapy. J Clin Oncol. 2018;36(9):850–8. 10.1200/JCO.2017.75.1644 29341833

[15] Gandhi L, Rodriguez-Abreu D, Gadgeel S, *et al*. Pembrolizumab plus chemotherapy in metastatic non-small-cell lung cancer. N Engl J Med. 2018;378(22):2078–92. 10.1056/NEJMoa1801005 29658856

[16] Langer CJ, Gadgeel SM, Borghaei H, *et al*. Carboplatin and pemetrexed with or without pembrolizumab for advanced, non-squamous non-small-cell lung cancer: a randomised, phase 2 cohort of the open-label KEYNOTE-021 study. Lancet Oncol. 2016;17(11):1497–508. 10.1016/S1470-2045(16)30498-3 PMC6886237 27745820

[17] Nakata J, Isohashi K, Oka Y, *et al*. Imaging assessment of tumor response in the era of immunotherapy. Diagnostics (Basel). 2021;11(6):1041. 10.3390/diagnostics11061041 PMC8226723 34198874

[18] Jia W, Gao Q, Han A, Zhu H, Yu J. The potential mechanism, recognition and clinical significance of tumor pseudoprogression after immunotherapy. Cancer Biol Med. 2019;16(4):655–70. 10.20892/j.issn.2095-3941.2019.0144. PMC6936240 31908886

[19] Paz-Ares L, Ciuleanu TE, Cobo M, *et al*. First-line nivolumab plus ipilimumab combined with two cycles of chemotherapy in patients with non-small-cell lung cancer (CheckMate 9LA): an international, randomised, open-label, phase 3 trial. Lancet Oncol. 2021;22(2):198–211. 10.1016/S1470-2045(20)30641-0 33476593

[20] Kurra V, Sullivan RJ, Gainor JF, *et al*. Pseudoprogression in cancer immunotherapy: rates, time course and patient outcomes. J Clin Oncol. 2016;34(15_suppl):6580.

